# Engineering a Photoautotrophic Microbial Coculture toward Enhanced Biohydrogen Production

**DOI:** 10.1021/acs.est.4c08629

**Published:** 2024-12-12

**Authors:** Minmin Pan, Rodrigo Amarante Colpo, Stamatina Roussou, Chang Ding, Peter Lindblad, Jens O. Krömer

**Affiliations:** † Department of Microbial Biotechnology, Helmholtz Centre for Environmental Research - UFZ, Leipzig 04318, Germany; ‡ Microbial Chemistry, Department of Chemistry-Ångström, Uppsala University, Box 523, Uppsala 75120, Sweden; § Department of Molecular Environmental Biotechnology, Helmholtz Centre for Environmental Research - UFZ, Leipzig 04318, Germany

**Keywords:** phototrophic community, H_2_ production, proteomics, metabolite exchange

## Abstract

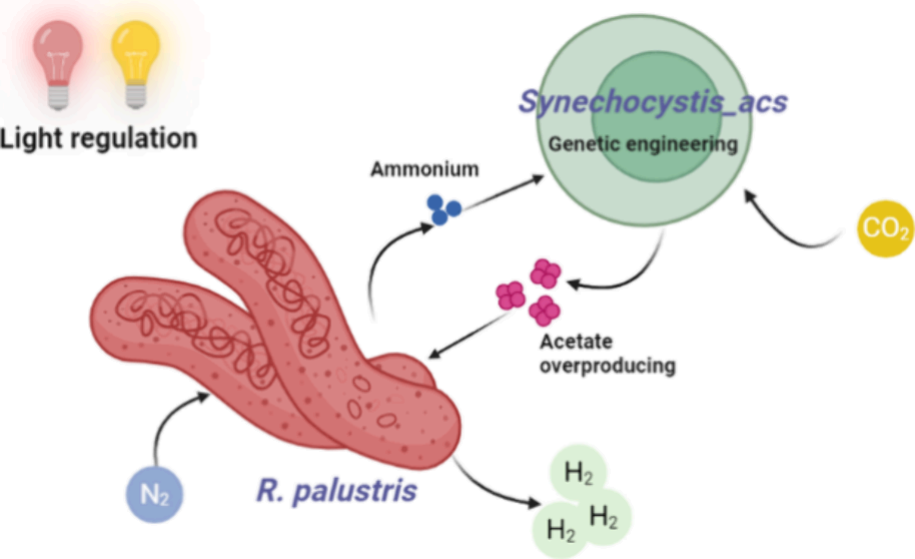

The application of synthetic phototrophic microbial consortia holds promise for sustainable bioenergy production. Nevertheless, strategies for the efficient construction and regulation of such consortia remain challenging. Applying tools of genetic engineering, this study successfully constructed a synthetic community of phototrophs using *Rhodopseudomonas palustris* (*R. palustris*) and an engineered strain of *Synechocystis sp* PCC6803 for acetate production (*Synechocystis_acs*), enabling the production of biohydrogen and fatty acids during nitrogen and carbon dioxide fixation. Elemental balance confirmed carbon capture and nitrogen fixation into the consortium. The strategy of circadian illumination effectively limited oxygen levels in the system, ensuring the activity of the nitrogenase in *R. palustris*, despite oxygenic photosynthesis happening in *Synechocystis*. When infrared light was introduced into the circadian illumination, the production of H_2_ (9.70 μmol mg^–1^) and fatty acids (especially C16 and C18) was significantly enhanced. Proteomic analysis indicated acetate exchange and light-dependent regulation of metabolic activities. Infrared illumination significantly stimulated the expression of proteins coding for nitrogen fixation, carbohydrate metabolism, and transporters in *R. palustris*, while constant white light led to the most upregulation of photosynthesis-related proteins in *Synechocystis_acs*. This study demonstrated the successful construction and light regulation of a phototrophic community, enabling H_2_ and fatty acid production through carbon and nitrogen fixation.

## Abstract 2

Engineered microbial coculture enables biohydrogen and fatty acid production using light energy and carbon/nitrogen fixation as inputs.

## 1 Introduction

Phototrophic microbial communities are commonly found in light-exposed environments.^1^ Such light-driven consortia contribute substantially to global primary production of organic compounds by fixing carbon dioxide and/or nitrogen gas.^2^ With humankind facing ever-growing energy demands and environmental problems,^3^ such synthetic phototrophic consortia may provide a promising alternative to current energy generation methods. These consortia can efficiently convert CO_2_ and N_2_ gases together with water and solar energy into products of bioenergy.^2,4^ In general, microbial consortia are also attracting more attention due to their ability for specialization and labor sharing, allowing them to achieve more complex and stable phenotypes compared to monocultures.^5,6^ Nevertheless, when trying to assemble a synthetic consortium outside the complex environmental constraints of a given habitat, a single strain in the consortium might become dominating, challenging the consortium stability. Thus, efficient strategies for maintaining strain balance and controlling the performance of consortia are major challenges.

Phototrophic microorganisms can be differentiated into water-splitting (oxygenic) and nonwater-splitting (anoxygenic) phototrophs. The former group includes cyanobacteria and microalgae, while the latter includes, for instance, purple nonsulfur bacteria (PNSB). *Rhodopseudomonas palustris* (*R. palustris*) is a prominent member of PNSB, which harbors diverse types of nitrogenases enabling N_2_ fixation under anoxic conditions with obligatory production of hydrogen gas.^7^ Despite being regarded as a promising candidate for biohydrogen and lipid production,^8,9^ the sufficient provision of organic carbon (e.g., acetate), which serves as the electron donor, is a current limitation.^10^ To address this issue, a coculture with cyanobacteria capable of effectively fixing atmospheric carbon dioxide into organic carbon,^11^ offers an alternative. With regard to N_2_ fixation, two types of cyanobacteria are distinguished: diazotrophic (N_2_ fixing) and nondiazotrophic cyanobacteria.^12^ The nondiazotrophic cyanobacteria might be advantageous in establishing a trophic-dependent coculture with *R. palustris*. Nevertheless, it remains elusive whether a coculture of such a consortium could be successfully established while maintaining sustainable production.

The conventional Haber–Bosch process (HBP), responsible for the annual production of 500 million tons of NH_3_, is highly energy-intensive, highlighting an urgent need for alternative technologies to improve sustainability.^13^ Notably, nitrogenase operates under mild conditions and is thus of great interest for addressing the challenge of cost-effective and sustainable ammonia synthesis. In addition to nitrogen fixation, nitrogenases are also considered as a route for hydrogen production; however, they are significantly inhibited by oxygen.^14^ In diazotrophic cyanobacteria, nitrogen fixation occurs in heterocyst cells under the consumption of organic compounds provided by the vegetative cells. Heterocyst cells themselves maintain an anaerobic environment by deactivating water splitting.^15^ Theoretically, when using a nondiazotrophic cyanobacterium, such as the model organism *Synechocystis* sp. PCC 6803 (hereafter *Synechocystis*),^16^ the role of the heterocyst would be taken over by *R. palustris*, which lacks the photosystem II protein complex, making water splitting impossible. To achieve a stable coculture of *Synechocystis* and *R. palustris*, balancing the CO_2_ and N_2_ fixation rates and the secretion of organic carbon and nitrogen while controlling the oxygen levels is critical. Unlike strictly anaerobic bacteria, *R. palustris* can switch among four trophic regimes and survive through both oxic and anoxic conditions,^7^ but a high oxygen tension inhibits N_2_ fixation and can lead to nitrogen starvation of the consortium. Under microaerobic conditions, however, energy can be provided through oxidative phosphorylation instead of photophosphorylation for nitrogenase activity.^17^ While the consortium might achieve a stable balance between the organisms through different ratios of the cell types themselves, an efficient strategy to regulate and limit the oxygen level in the consortium is crucial to achieving the desired pathway activities.

Light conditions play a significant role in regulating the performance of phototrophs. Allowing for dark respiration of microbes is an effective way to metabolize oxygen, thus ensuring anoxic or oxygen-depleted conditions for H_2_ production.^18^ Complete microbial oxygen reduction would simply undo the water splitting by producing water, and no hydrogen would be generated. However, lowering oxygen tension around the PNSB enough to enable the activity of nitrogenases or hydrogenases would complement physical oxygen removal from the reactor, for instance, through gas stripping. As a typical cyanobacterium, *Synechocystis* contains a series of pigments, e.g., chlorophylls and phycobiliproteins, enabling the utilization of visible light for photosynthesis.^19^ In addition to visible light, *R. palustris*, however, also harbors a light-harvesting complex (bacteriochlorophylls) that can absorb light in the near-infrared spectrum (NIR, 800–900 nm).^20^ This difference should allow, on the one hand, the possibility to create a coculture that uses a broader light spectrum than cyanobacteria alone, increasing photon efficiency, and, on the other hand, should provide a strategy for selectively providing light energy in the coculture to individual strains. This could facilitate the balancing of strain abundance and regulate metabolic activities within the coculture by changing light conditions.

In this study, we constructed a coculture consisting of cyanobacteria and PNSB through light-dependent regulation. We cocultivated *R. palustris* with either the wild type of *Synechocystis* sp. PCC 6803, or an engineered strain, *Synechocystis_acs* (an acetate overproducing strain). Various light regulation strategies, including constant illumination, circadian light-dark illumination, and circadian light-infrared illumination, were employed. These strategies facilitated trophic dependence through carbon and nitrogen assimilation and allowed for the regulation of coculture growth. The coculture enabled biohydrogen production in a light-based system feeding on CO_2_ and N_2_, highlighting the potential of controlling a phototrophic community.

## 2 Materials and Methods

### 2.1 Materials

The purple nonsulfur bacterium*Rhodopseudomonas palustris* ATCC BisB5 (hereafter *R. palustris*) was purchased from ATCC (American Type Culture Collection, Manassas, Virginia, USA). The cyanobacterium *Synechocystis* sp. PCC 6803 (hereafter *Synechocystis*) and engineered strain *Synechocystis* sp. PCC 6803 WT_Δacs_PKPa (hereafter *Synechocystis_acs*), which overproduces acetate, were used. Briefly, the plasmid used for the genetic engineering was based on a pEERM vector.^21^ A knockout of the *acs* gene (encoding acetyl-CoA synthetase from acetate) with a parallel insertion of the PKPa gene (encoding phosphoketolase, which synthesizes acetyl-phosphate from Xu5P and F6P) was conducted to overproduce acetate in *Synechocystis_acs*. The designed sequences were amplified from the genome of *Synechocystis* and then joined with the phosphoketolase gene (PKPa) under the control of the PtrcRiboJ promoter.^22^ The details of the genetic modification are described in Supporting Information Text 1. *R. palustris* was precultured in a modified M27 preculture medium (Table S2), while *Synechocystis* and *Synechocystis_acs* were grown in BG11 and BG11 plus (with 50 mg/L kanamycin, Table S3) media, respectively. All reagents (analytical grade) were purchased from Sigma-Aldrich. Ultrapure Water (18 MΩ·cm^–1^) was applied for medium preparation.

### 2.2 Cultivation Procedures

Cultivation experiments were performed in triplicate using sealed (initially anaerobic) cultivation flasks (120 mL, Wheaton) with a working volume of 30 mL. Strains were stored in glycerol stocks (M27 with 25% glycerol and BG11 with 7% DMSO for *R. palustris* and *Synechocystis*, respectively) at −80 °C. For reactivation, glycerol stocks were streaked out on corresponding agar plates (with 1.5% agar). Single colonies were transferred to the corresponding liquid medium, separately. Inocula of *R. palustris* and *Synechocystis* (or *Synechocystis_acs*) were harvested by centrifugation (4000*g*, room temperature, 5 min). The cells were washed three times by resuspension in fresh medium (using modified M27 or BG11 medium, respectively), followed by centrifugation. The washed and resuspended cells (in 0.5 mL modified M27) were then used to inoculate the main cultivation to achieve initial cell densities of 4.3 ± 0.3 × 10^7^ and 4.0 ± 0.3 × 10^6^ cells mL^–1^ in each treatment, respectively. The final cell numbers of inoculum from each strain were decided by pre-experiment to ensure a simultaneous growth and efficient production of H_2_.

In this study, cold white light (Osram Lumilux, 18 W, Germany) and infrared light (Synergy 21, 24 W, 850 nm, LED, Germany) were applied. Three light conditions were employed: (i) 24 h day^–1^ white light (W), (ii) 16 h day^–1^ white light followed by 8 h day^–1^ darkness (WD), and (iii) 16 h day^–1^ white light followed by 8 h day^–1^ infrared light (WI). To differentiate the impact of light quality and coculture effects, a total of six triplicate experiments were conducted. First, the light condition WI was studied in monocultures of *Synechocystis_acs* (S_acs_-WI), monocultures of *R. palustris* (R-WI), cocultures of *R. palustris* with *Synechocystis* (C_wt_-WI), and cocultures of *R. palustris* with *Synechocystis_acs* (C–WI). Second, the cocultures of *R. palustris* with *Synechocystis_acs* were further explored under light conditions L (C–W) and LD (C-WD). All cultivations were performed in a modified M27 medium (Table S2).

All bottles were placed on an orbital shaker (25 mm shaking orbit, HT multitron Pro, Infors, Switzerland) with a shaking speed of 100 rpm and an illumination of 50 μmol photons m^–2^ s^–1^ at 30 °C. Inoculation and daily sampling were all conducted on a clean bench.

### 2.3 Analysis of Cell Growth

To differentiate the growth of each strain in the consortium, a flow cytometer (CytoFLEX, Beckman Coulter, Germany) was used for cell counting. Specifically, a blue laser (488 nm) with an optical filter mount of 780/60 (Gain: 500) and 660/20 (Gain: 200) was set for the cell counting of *R. palustris* and *Synechocystis*/*Synechocystis_acs*, respectively. A SYTO 9 stain (Thermo Fisher Scientific) was used for staining the cells before measurement. The software CytExpert was used for machine operation and data analysis.

Total cellular dry weights were estimated by using correlation factors between dry weights and cell numbers. Specifically, 10.1 mL of each monoculture or coculture was sampled for detection of cell numbers with the flow cytometer (0.1 mL), and the corresponding dry weights (10 mL) were determined after lyophilization of the cell pellets (after centrifugation, pellets were washed twice with modified M27 medium and once with milli-Q water, then, lyophilized. The results were used to obtain the following correlations between cell counts and dry weight (eqs 1 and 2):
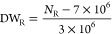
1
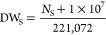
2Where DW_R_ and DWs are the dry weights (mg L^–1^) and *N*_R_ and *N*_S_ are the cell numbers (cells per mL culture medium) of *R. palustris* and *Synechocystis*, respectively.

### 2.4 Quantification of Gas Composition

A gas chromatograph (GC, TRACE 1310, Thermo Fisher Scientific) coupled with a TG-Bond MIsieve column (film thickness: 0.30 μm, length: 30 m, diameter: 0.32 mm, Thermo Scientific) was employed to detect H_2_ and O_2_ in the headspace. A sample of 0.1 mL was injected using a GC manual gastight syringe (0.1 mL, Thermo Fisher), with a column flow of 2 mL min^–1^ with argon as the carrier gas at an oven temperature of 75 °C. Detection was performed with a thermal conductivity detector (TCD) at a detector temperature of 100 °C and a filament temperature of 200 °C. The total run time was 2.4 min. Sampling was conducted once a day at the start of each white illumination phase. This timing and frequency were determined based on preliminary experiments, which frequently monitored H_2_ and O_2_ production across treatments to capture representative gas fluctuation trends.

### 2.5 Analysis of Elemental Balances

The carbon balance was calculated with the dominant carbon-containing compounds in the supernatant including acetate, citrate, cysteine, urea, bicarbonate, and biomass. The nitrogen balance was calculated with ammonium, urea, cysteine, and biomass. The sulfur balance was calculated with sulfate, cysteine, thiosulfate, and biomass.

For the quantification of positively charged compounds, different analytical methods were employed. Ammonium concentrations were analyzed using a cation chromatograph (Dionex Integrion HPIC system, Thermo Fisher, USA) with a flow rate of 0.25 mL min^–1^ and a total run time of 30 min. Gradient elution was performed using an eluent generator cartridge (EGC 500 MSA cation, 074535, Thermo Fisher) with a concentration gradient, as described in Table S4. A conductivity detector with a data collection rate of 5.0 Hz was used for compound detection.

Negatively charged compounds, such as acetate, citrate, bicarbonate, sulfate, and thiosulfate, were quantified with IC-MS, while cysteine and urea were detected with HPLC-MS. All negatively charged compounds were measured on days 0 and 4. When bicarbonate was measured with IC-MS, the eluents were first flushed with nitrogen gas. The pH of all supernatant samples was adjusted to 12 (with 1 M NaOH) and filtered (0.22 μm, 13 mm diameter, Whatman, UK) before detection. After allowing the samples to equilibrate for 120 min, the bicarbonate concentrations were measured as carbonate.

An ion-chromatograph (ICS-6000, organic acid column, Dionex IonPac CS19-4 μm, RFIC, 2 × 250 mm) coupled with a mass spectrometer (Orbitrap Exploris 240 Mass Spectrometer, Thermo Fisher) was used for quantifying the negatively charged compounds mentioned above. Specifically, for the IC-MS run, the flow rates of the makeup pump, regenerate pump, and main pump (ICS-6000) were 0.15 mL min^–1^ (methanol), 0.5 mL min^–1^ (Milli-Q water), and 0.38 mL min^–1^, respectively. The column and detector/chromatography compartment temperatures were 30 and 20 °C, respectively. An EGC 500 KOH cartridge (eluent) was employed to generate the flow gradient, as described in Table S5. Besides, an HPLC system (Vanquish HPLC, Thermo Fisher; with a HILIC column, InfinityLab Poroshell 120 HILIC-Z, 2.1 × 150 mm, 2.7 μm, Agilent) coupled with the above-mentioned Orbitrap was applied as the HPLC-MS system to quantify cysteine and urea. A 10 mM ammonium formate solution (pH = 3) either in Milli-Q water (eluent A) or in 90% acetonitrile (eluent B) was employed with the flow gradient, as described in Table S6. The column temperature was set to 25 °C, and UV absorbance was monitored at 220 nm.

The MS scan range was set as 40–900 with a detection resolution of 15,000. For both IC/HPLC-MS systems, the H-ESI ion source was applied with static spray voltage modes of 3500 V (positive ion) and 2500 V (negative ion). Temperatures were set to 325 °C for the ion transfer tube and 300 °C for the vaporizer. A full scan method with data-dependent MS^2^ detection (secondary MS) was applied. Software Chromeleon Console (version 7) and Compound Discoverer 3.3 were used for data analysis.

### 2.6 Measurement of Fatty Acids

For the detection of fatty acids, 5 mL of samples from each experiment was collected on day 4. Cells were separated from the broth using fast filtration (cellulose nitrate filter, Whatman CN, 47 mm/0.22 μm) and washed twice using a modified M27 medium. The washed filters with the biomass were then transferred into 5 mL centrifuge tubes and snap-frozen with liquid nitrogen for 5 min. After 3.5 mL of cold extraction solution (1:1:5 MeOH, CHCl_3_, and Milli-Q water, with 0.125 mmol L^–1^ nonadecanoic acid as an internal standard in the CHCl_3_ layer) and glass beads were added into the centrifuge tubes, cells were then disrupted with sonication (ice bath, 10 min). After centrifugation (17,000*g*, 7 °C), the CHCl_3_ layer was taken for further detection of fatty acids.

Next, 0.5 mL of 5% acetyl chloride in MeOH was added to the samples and esterified for 30 min at 90 °C. Afterward, 0.5 mL of hexane was added and vortexed for 10 s to extract all fatty acid methyl esters (FAMEs). Of the hexane phase, 0.4 mL was transferred to GC vials for measurement. FAMEs from C8 to C24 were measured by using a gas chromatography system (TRACE 1310, Thermo Fisher Scientific) coupled with a single quadrupole mass spectrometer (ISQ 7000, Thermo Fisher Scientific). A TG-Bond Mlsieve column (length: 30 m; diameter: 0.32 mm; film thickness: 0.30 μm, Thermo Scientific) was employed. The temperatures of the inlet and the GC oven (start point) were 250 and 60 °C, respectively, with an inlet flow of 1 mL min^–1^. The specific GC-MS oven temperature gradient is described in Table S7. For each measurement, a total run time of 42 min was set. The specific calculations of fatty acid titer and content are described in Supplementary Text 2.

### 2.7 Proteome Analysis

Samples for proteomics were taken on day 4 of each experiment. Cell pellets were collected by centrifugation at 4500*g* for 10 min at 7 °C and then resuspended in ammonium bicarbonate buffer (100 mM). Cells were then disrupted using three cycles of freeze/thaw (−80 and +40 °C for 2 and 1 min, respectively). Five microliter of bovine serum albumin (BSA, 20 ng μL^–1^) was used as an internal standard for each proteomic sample. For denaturing proteins, an equal volume of 10% (w v^–1^) sodium dexycholate was added to all samples. Then, 12 mM DTT (final concentration) was added to reduce proteins at 37 °C for 45 min. 40 mM iodoacetamide (final concentration) was used for protein alkylation at room temperature for 45 min (in the dark, with shaking). Samples were diluted to 1% (w v^–1^) sodium deoxycholate with 100 mM ammonium bicarbonate. For trypsin digestion, 5 μL of reductively methylated trypsin (0.1 μg μL^–1^) (Promega, Madison, USA) was added to each sample and incubated overnight at 37 °C. Neat formic acid was added to a final concentration of 2% (v v^–1^) to stop digestion, followed by twice centrifugation at 16,000 × *g* (10 min, 4 °C) to remove any precipitation. C18 ziptips with 100 μL volume (Millipore, Merck, USA) were employed for peptide desalting, and desalted peptides were reconstituted in 0.1% v v^–1^ formic acid after vacuum drying.

A nano-LC system (Dionex Ultimate 3000RSLC, Thermo Scientific) coupled with an Orbitrap Fusion Tribrid mass spectrometer (Thermo Scientific) was employed for the detection of peptides after separation on an Acclaim PepMap 100 C18 column (100 Å pore size, 3 μm particle size, 75 μm × 250 mm, Thermo Scientific) with a flow rate of 0.3 μL min^–1^ and a column oven temperature of 35 °C. A solution of 0.1% v v^–1^ formic acid in Milli-Q water and 0.08% v v^–1^ formic acid in water/acetonitrile (20:80%) served as mobile phases A and B, respectively. Gradient elution was performed as described in Table S8.

Mass spectrometry analysis was performed in positive mode according to a previous study.^23^ Briefly, both MS1 and MS2 scans were conducted. The specific parameters of the Orbitrap were NSI ion source type; method duration of 140 min; 2400 V positive ion and 600 V negative ion of spray voltage; ion transfer tube temperature of 275 °C; detector type Orbitrap; scan range 350–2000 *m*/*z*; AGC target 400,000; RF lens 60%; filter MIPS; MIPS mode peptide. Only ions with a charge state between 2 and 4 were selected for fragmentation. Proteome Discoverer (version 2.4, Thermo Fisher Scientific) was used for analyzing the acquired raw data. SequestHT was used to search all MS2 spectra against the FASTA files containing all protein sequences in the genome of *Synechocystis* sp. PCC 6803 Kazusa (for *Synechocystis_acs*, the protein sequence of PKPa from **Pseudomonas aeruginosa** was manually inserted) and *R. palustris* BisB5. Intensity-based label-free quantification using the Minora node was used for calculating the protein and peptide abundance.

### 2.8 Gene Ontology Analysis, Calculation, and Statistical Analysis

The gene ontology (GO) analysis was conducted to incorporate information on differentially expressed proteins and enrichment metrics simultaneously (as described in Supplementary Text 3). Briefly, after mapping the identified proteins to UniProt IDs, Fisher’s exact test was applied to combine *p*-values of protein abundance with the same GO annotation, identifying expressions with significance. This was followed by the Benjamini–Hochberg procedure and hypergeometric test to identify the false discovery rate (FDR) and enrichment. R Studio (version 3.6) and Origin 8.5 (OriginLab, Northampton, USA) were used to analyze and plot the data, respectively.

## 3 Results and Discussion

To achieve a trophic dependency and simultaneous growth of each strain in the coculture, acetate, and yeast extract were replaced with bicarbonate, and the major nitrogen source was changed to N_2_ compared to the original M27 medium. The provision of bicarbonate meant that the coculture relied on carbon fixation by *Synechocystis*. Nitrogen was initially provided in the form of urea at a low concentration. This was selected as an initial booster to start the growth of the community. The amount of urea provided would become limiting soon after some initial growth, making the coculture reliant on N_2_ fixation by *R. palustris*.

### 3.1 Growth of the Consortium

**Figure 1:**
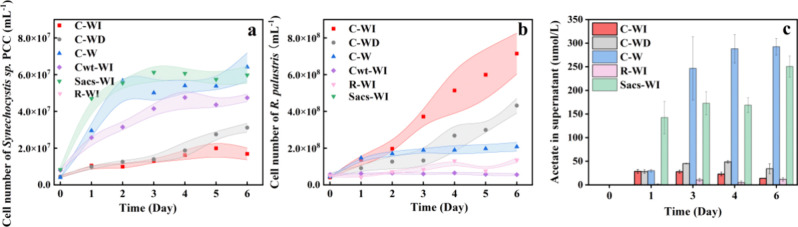
Growth (cell number) of (a) *Synechocystis.* sp and (b) *R. palustris* under different treatments and (c) concentration of acetate in supernatant. R: *R. palustris*; S: *Synechocystis* wild type; Sacs: *Synechocystis_acs*; -L: group of constant white light illumination; -LD: group of circadian illumination of light and dark; -LR: group of circadian illumination of light and infrared.

Under circadian illumination of white and infrared light (WI), *Synechocystis* in the coculture (C-WI) achieved similar growth compared to the monoculture of *Synechocystis_acs* (Sacs-WI). However, much slower growth was observed for *Synechocystis_acs* in the coculture (C-WI, Figure 1a). Correspondingly, due to the lack of an organic carbon source, the growth of *R. palustris* in both monoculture (R-WI) and coculture with *Synechocystis* (C_wt_-WI) was significantly inhibited (Figure 1b). Notably, in the presence of *Synechocystis_acs*, *R. palustris* (C-WI) achieved significantly higher growth (5.3 and 12.8 times) compared to that of the R-LR and R + S-LR, respectively. This indicates a potential provision of organic compounds from *Synechocystis_acs* to *R. palustris* in the coculture. Nevertheless, light conditions affected the performance of the cocultures strongly. An illumination with a light and dark cycle promoted the simultaneous growth of both strains (with slow growth rates in C-WD); however, a constant illumination resulted in a dominant growth of *Synechocystis_acs*, thus further inhibiting the *R. palustris* (C-W). Notably, the coculture with *Synechocystis_acs* under a circadian rhythm of white and infrared light promoted the growth of *R. palustris* the most. The coculture promoted a constant and longer exponential growth of *R. palustris* than the monoculture fed with standard organic carbon sources, e.g., acetate and malate,^24^ indicating a promising potential to use the proposed coculture for generating sustainable growth of *R. palustris*.

The culture medium used in this study was acetate-depleted. As a critical compound, acetate could be overproduced and released by the engineered strain (*Synechocystis_acs*) used in the present study, and serve as an organic carbon source for growth and/or H_2_ production of *R. palustris*.^8^ The results show that the cultures with rapid growth of *Synechocystis_acs* but limited growth of *R. palustris* (or without *R. palustris*) accumulated much higher acetate concentrations in the supernatant than the other cultures (up to 292.3 and 250.6 μmol L^–1^ acetate in C-W and Sacs-WI, respectively, Figure 1c). In contrast, when *R. palustris* exhibited significant growth, less acetate was detected in the supernatant (up to 34.4 and 13.8 μmol L^–1^ acetate in C-WD and C-WI, respectively), indicating a potential rapid acetate consumption that supported the growth of *R. palustris*.

### 3.2 Gas Composition and Enriched Products of Renewable Energy Carrier

**Figure 2:**
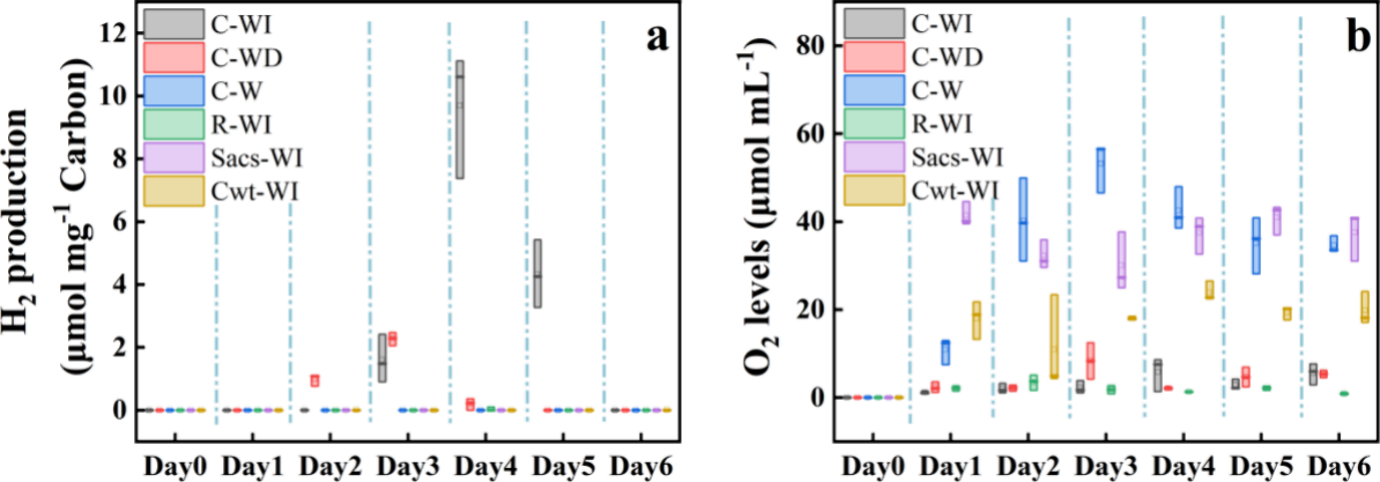
Gas composition in headspace with different treatments. (a) Produced hydrogen gas yield per mg fixed carbon and (b) oxygen gas levels.

As shown in Figure 2, the coculture of *R. palustris* with *Synechocystis_acs* under light-dark illumination (C-WD) started producing hydrogen gas at the earliest and reached the maximum hydrogen yield of 2.27 ± 0.21 μmol mg^–1^ at day 3 (the hydrogen production yield per milligram of fixed carbon). Meanwhile, the coculture under circadian white and infrared illumination accumulated hydrogen later (from day 3) but reached the highest hydrogen production (up to 9.70 ± 2.03 μmol mg^–1^, C-WI) among all treatments (Figure 1a).

*R. palustris* encodes diverse nitrogenases responsible for fixing nitrogen gas into ammonia with the obligatory byproduct of hydrogen.^8^ The various enzymatic options of nitrogenases in *R. palustris* help to mitigate the inhibitions under adverse conditions, such as limited nutrients,^25^ thus ensuring high efficiency of H_2_ production in practice. Besides, *R. palustris* can utilize a wide range of organic carbon sources for H_2_ production,^10^ which broadens the application scenarios for biohydrogen production. However, nitrogenases and hydrogenases are sensitive to oxygen.^14^ Therefore, limiting the oxygen level in the system is critical for the regulation of hydrogen gas production. In this study, circadian illumination effectively maintained oxygen at depleted levels (C-WI and C-WD, Figure 1b). The respiration during the dark or infrared phase allowed the microbes to partially metabolize oxygen.^18^ The reduced oxygen levels were low enough to enable hydrogen production. In contrast, even with low oxygen content in the monoculture of *R. palustris* (R-WI), no hydrogen gas was produced due to the lack of organic carbon sources that serve as electron donors.

Hydrogen production via nitrogenase is an energy-intensive process, with nitrogenase competing for ferrodoxin and protons, thereby diverting these resources from other metabolic pathways. This leads to an overall competition for energy, protons, and electrons, impacting cellular growth, carbon storage, and various metabolic functions.^24,26^ However, circadian illumination provides *R. palustris* with an opportunity to balance resource allocation across different metabolic pathways, potentially improving the overall production efficiency. In *R. palustris*, light-driven reactions facilitate NADH production and electron transfer. However, electrons are ultimately sourced from the oxidation of organic compounds without any water-splitting process taking place. The electrons can be directed to three primary electron sinks, including nitrogen fixation (with H2 production), anabolic pathways, or storage metabolism.^27^ During white light exposure, *R. palustris* consumed organic carbon in the coculture, while circadian dark phases channeled electrons primarily toward biomass and likely to storage metabolism. In contrast, infrared light phases under circadian conditions appear to preferentially allocate electron flow toward N_2_ fixation with the nitrogenase producing H_2_ as a byproduct. Consequently, the C-WI treatment achieved substantially higher H_2_ yields compared with the C-WD treatment.

Future studies aim at a comprehensive electron balance analysis that could provide deeper insights into the mechanisms by which varying light regimes influence electron allocation across metabolic pathways.

**Figure 3:**
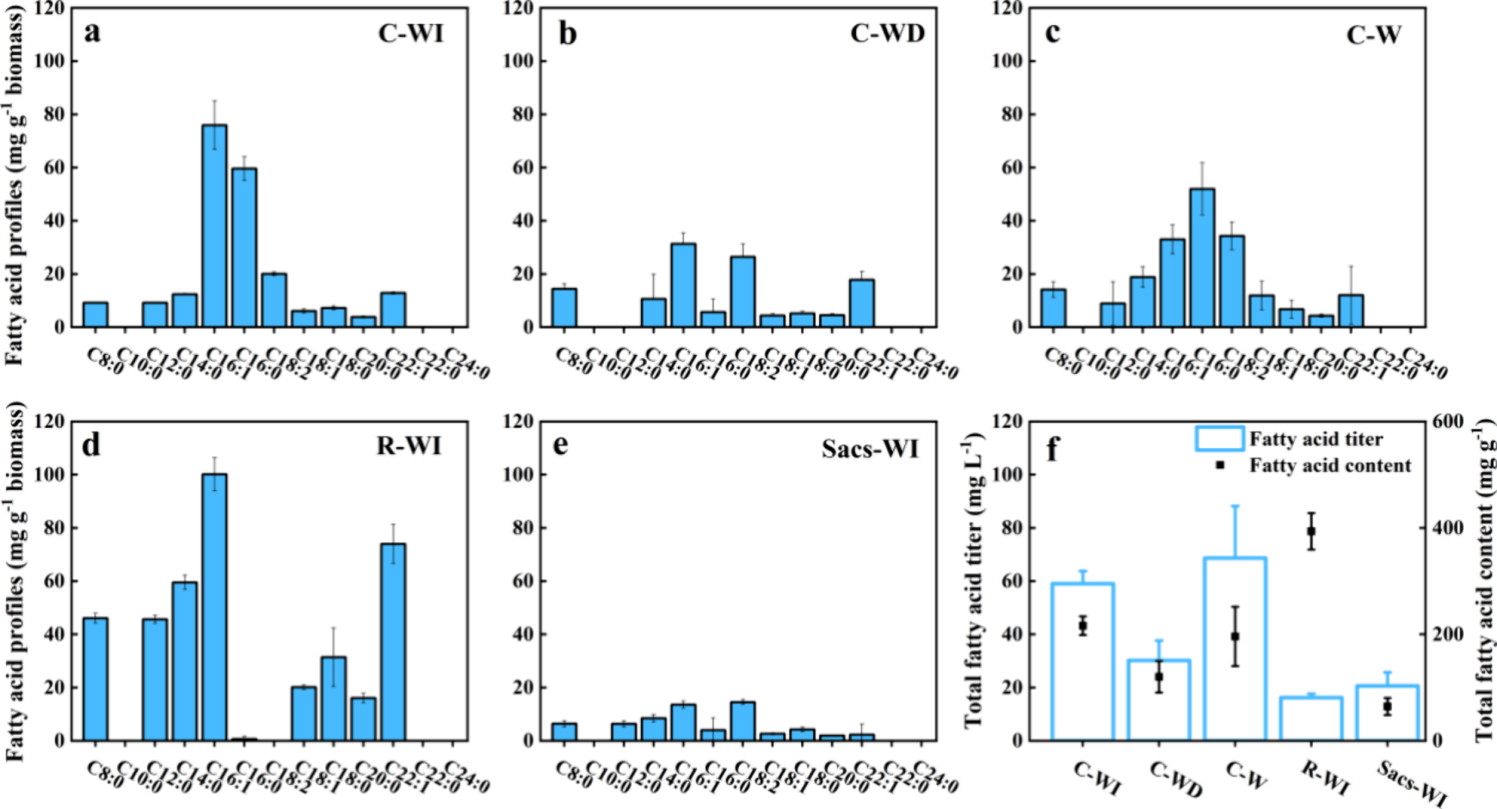
C8–C24 fatty acid profiles of (a) C-WI, (b) C-WD, (c) C-W, (d) R-WI, and (e) Sacs-WI. (f) Total fatty acid content (left) and yield (right) covering C8–C24 in each treatment.

In addition to H_2_, fatty acids also serve as critical renewable energy carriers.^28^ Results of fatty acid profiling suggested a lower fatty acid content (C8–C24) in *Synechocystis_acs* compared to *R. palustris* (Figure 3d,e). Thus, the monoculture of *Synechocystis_acs* generated only 20.6 mg L^–1^ of fatty acids out of the total biomass. Conversely, even though all three cocultures produced significantly higher total fatty acid titers compared to the monocultures, substantial differences in total fatty acid titers were observed among the three cocultures (Figure 3f). The coculture of *R. palustris* with *Synechocystis_acs* under constant illumination (C-W) and circadian white-infrared illumination (C-WI) gained significantly higher total biomass compared to the C-WD group, resulting in higher total fatty acids titers (68.7 and 59.0 mg L^–1^ respectively, C8–C24) in C-W and C-WI treatments. Although C-WI promoted greater growth of *R. palustris* (154 mg L^–1^) than C-W (61 mg L^–1^), the changes in the fatty acid contents led to no significant difference in total fatty acid titer between the two treatments.

Regarding the fatty acid profile, the coculture with constant white light and circadian white-infrared conditions triggered the highest accumulation of C16–C18 fatty acids (mainly C16:1, C16:0, and C18:2) in the final biomass. The unsaturated fatty acid palmitoleic acid (POA, C16:1) has demonstrated the ability to prevent a series of diseases, e.g., stroke and diabetes.^29^ In addition, the long-chain unsaturated fatty acids, including oleic acid (C18:1) and linoleic acid (C18:2), have been described as attractive antibacterial agents, and thus serve as key antimicrobial food additives.^30^ Consequently, the cocultures (C-WI and C-W) demonstrate their potential for enhancing the production of both biofuels and valuable chemicals through carbon capture. Such fatty acids-derived biofuels (e.g., via transesterification and esterification) even have a higher energy density and are more compatible with current infrastructure when compared to other forms of renewable energy.^31^

### 3.3 Carbon and Nitrogen Balancing

**Figure 4:**
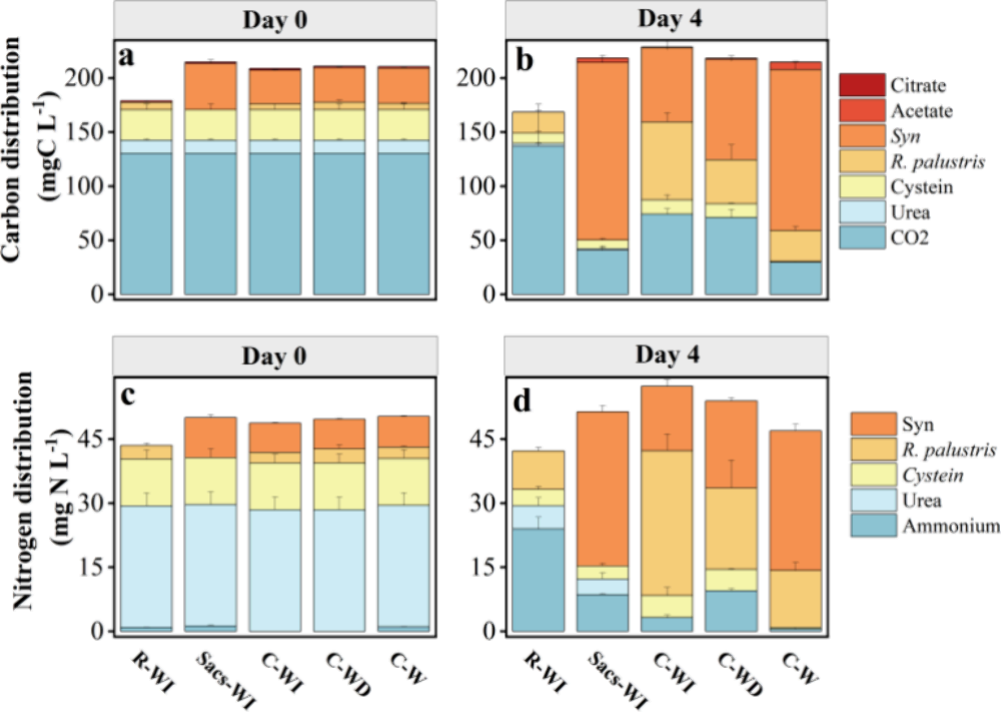
Elemental distribution for (a) carbon at day 0, (b) carbon at day 4, (c) nitrogen at day 0, and (d) nitrogen at day 4.

The initial carbon source in the system was mainly bicarbonate (60.6–72.8%, detected as carbonate), cysteine (13.2–15.8%), urea (5.7–6.8%), and the cellular inoculum. Under the chosen cultivation conditions, bicarbonate could not be directly metabolized by *R. palustris*,^7^ therefore, neither significant consumption of bicarbonate, nor significant growth of *R. palustris* was observed in the monoculture of *R. palustris* (R-WI, Figure 4a,b). In contrast, the photosynthesis of *Synechocystis_acs* in monoculture (Sacs-WI) rapidly fixed carbon from bicarbonate (carbon content reduced from 60.6 to 18.2%) and redirected the carbon flow toward biomass (73.4%) as well as excreted acetate (1.8% accumulated in the supernatant after consumption by *R. palustris*) at day 4. In comparison, the coculture with constant light (C-W) generated a similar flow from carbon fixation toward biomass of *Synechocystis_acs* (67.6%), but with higher excreted acetate (3.1% left after consumption). Notably, in both cocultures with circadian illumination, a smaller proportion of the fixed carbon was retained within the biomass of *Synechocystis_acs* (28.4% and 42.2% in C-WI and C-WD, respectively) compared to the monoculture. In these conditions, a larger proportion of the fixed carbon was incorporated into the biomass of *R. palustris* (33.6 and 20.4% in C-WI and C-WD, respectively), indicating an organic carbon supply from *Synechocystis_acs* to *R. palustris*.

Nitrogen plays a critical role in the synthetic consortium. Urea served as the primary nitrogen source in the system, accounting for 65.4% of the total nitrogen (Figure 4c,d). By day 4, nitrogen from urea was depleted in all coculture treatments, while residual nitrogen was still observed in both monocultures of *R. palustris* and *Synechocystis_acs*. Nitrogen in the form of urea can be rapidly transformed into ammonium through urease and further metabolized by *Synechocystis_acs* and *R. palustris*. Subsequently, the biomass of *Synechocystis_acs* and/or *R. palustris* became the dominant sink of nitrogen, indicating nitrogen uptake and supporting further growth of biomass (Figure 4d). Notably, in the cocultures, illumination conditions significantly regulated the final sink of nitrogen, in which circadian illumination of white/infrared promoted the highest accumulation of nitrogen in the biomass of *R. palustris* (59.1%, R + Sacs-LR), while constant white light led to the lowest (28.5%, C-W). In all three cocultures, the ammonium detected in the supernatant increased initially (from day 0 to day 2) and then decreased at day 4 (Figure S2). Meanwhile, in the monocultures, the ammonium levels increased and remained stable until day 4. The ammonium in the supernatant could either be hydrolyzed from urea via urease or be released by *R. palustris* through nitrogen fixation (nitrogenase). The nitrogen balances closed at 117.6, 108.6, and 93.3% on day 4 compared to day 0 in C-WI, C-WD, and C-W, respectively, indicating fixation of gaseous nitrogen from the headspace into the cocultures under WI and WD illumination. The coculture presents a promising alternative in sustainable ammonia synthesis, offering a viable substitute for conventional HBP. This nitrogen fixation also led to obligatory hydrogen production, especially in the coculture of C-WI.

Interestingly, thiosulfate was observed in the coculture of *R. palustris* with *Synechocystis*_acs under both constant illumination (C-W) and circadian illumination of light-infrared (C-WI) at day 4 (Figure S1b). Thiosulfate can serve as an electron donor for the nitrogenase-catalyzed H_2_ production by *R. palustris*.^32^ Therefore, in the coculture, both acetate and thiosulfate could potentially promote H_2_ production by donating electrons.

### 3.4 Response of Proteome to Coculture and Light Regulation

**Figure 5:**
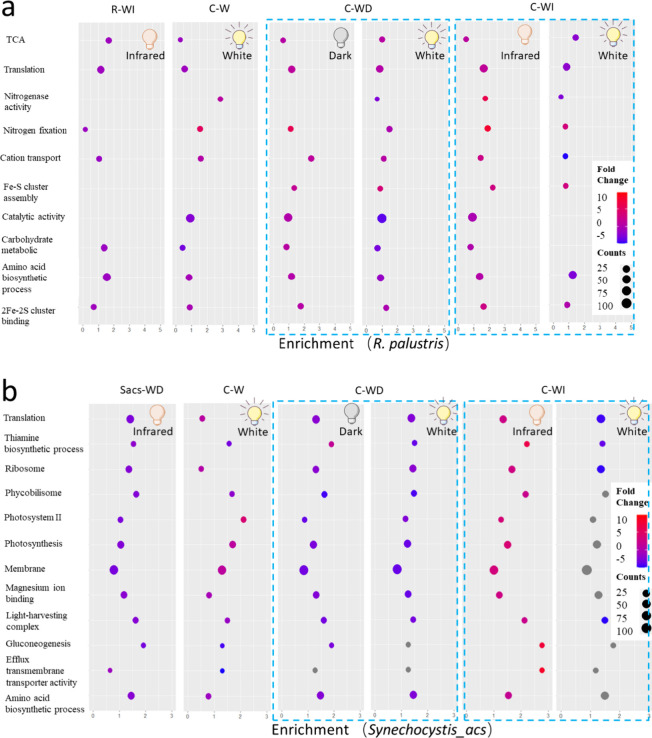
Gene ontology (GO) enrichment analysis and pathway significance analysis of different treatments for (a) *R. palustris* and (b) *Synechocystis_acs.* Yellow bulb: white light period; gray bulb: dark period; red bulb: infrared period; R: *R. palustris*; Sacs: *Synechocystis_acs*; -W: group of constant white light illumination; -WD: group of circadian illumination of light and dark; -WI: group of circadian illumination of light and infrared. The fold changes of *R. palustris* and *Synechocystis_acs* were compared to the light period of the single culture of *R. palustris* (in R-WI treatment) and the single culture of *Synechocystis_acs* (in Sacs- WI treatment), respectively. Counts refer to protein number that has been detected. Gray bubbles refer to the GO terms with no significant difference (*p* > 0.05).

To better understand the underlying metabolic processes in the cocultures under varying illumination conditions, a proteomics analysis was conducted. In general, among all treatments, *R. palustris* in the coculture during infrared periods (infrared C-WI) gained the most enrichment of gene ontology (GO) terms (Figure 5a), meaning that significant changes of metabolic activities (both up- and downregulations) took place when the cocultures were exposed to infrared illumination in the coculture compared to the single culture. In the coculture, compared to the white light periods (white C-WI), during infrared illumination (infrared C-WI), the proteins encoding the biological processes of nitrogen fixation, monatomic cation transmembrane transport, carbohydrate metabolic process, translation, and Fe–S cluster assembly were significantly enriched and upregulated. Meanwhile, the molecular functions, e.g., nitrogenase activity and 2Fe–2S cluster binding also showed an upregulated trend. The carbohydrate metabolic processes, e.g., acetate metabolism, ensure the electron balance for hydrogen production.^8^ Besides, the Fe–S cluster is closely related to the formation of hydrogenase and nitrogenase.^33^ With sufficient electron donors (from acetate metabolism), the upregulated nitrogen fixation could lead to obligatory H_2_ production.^8^ This indicated a generally stimulated activity of *R. palustris* in the coculture, particularly toward H_2_ production, electron/substrate transportation, and biomass accumulation during infrared illumination. Unlike C-WI, nitrogenase activity-related proteins in C-WD were exclusively detected during white illumination, indicating that a different H_2_ production period occurred in C-WD (during white illumination) compared to C-WI (during infrared illumination). The presence of white light led to competitive interactions between *R. palustris* and *Synechocystis_acs*, significantly suppressing the carbohydrate metabolism and amino acid biosynthesis of *R. palustris* in both C–WI and C-WD. Conversely, the circadian cycles of illumination, which included dark or infrared periods, provided *R. palustris* with opportunities for metabolism.

For *Synechocystis_acs*, the highest enrichment and upregulation of photosynthesis and photosystem II were achieved under continuous illumination (C-W). This accounts for the highest growth (Figure 1a) and carbon fixation (Figure 4b) observed in *Synechocystis_acs*, which also led to elevated O_2_ levels (Figure 2b). Interestingly, the amino acid biosynthesis and efflux transmembrane transporter activity^34^ were more stimulated in the coculture during infrared illumination (C-WI), indicating a potential higher cellular growth of *Synechocystis_acs* and increased substrate release and supply (e.g., acetate) to *R. palustris* during infrared periods.

**Figure 6:**
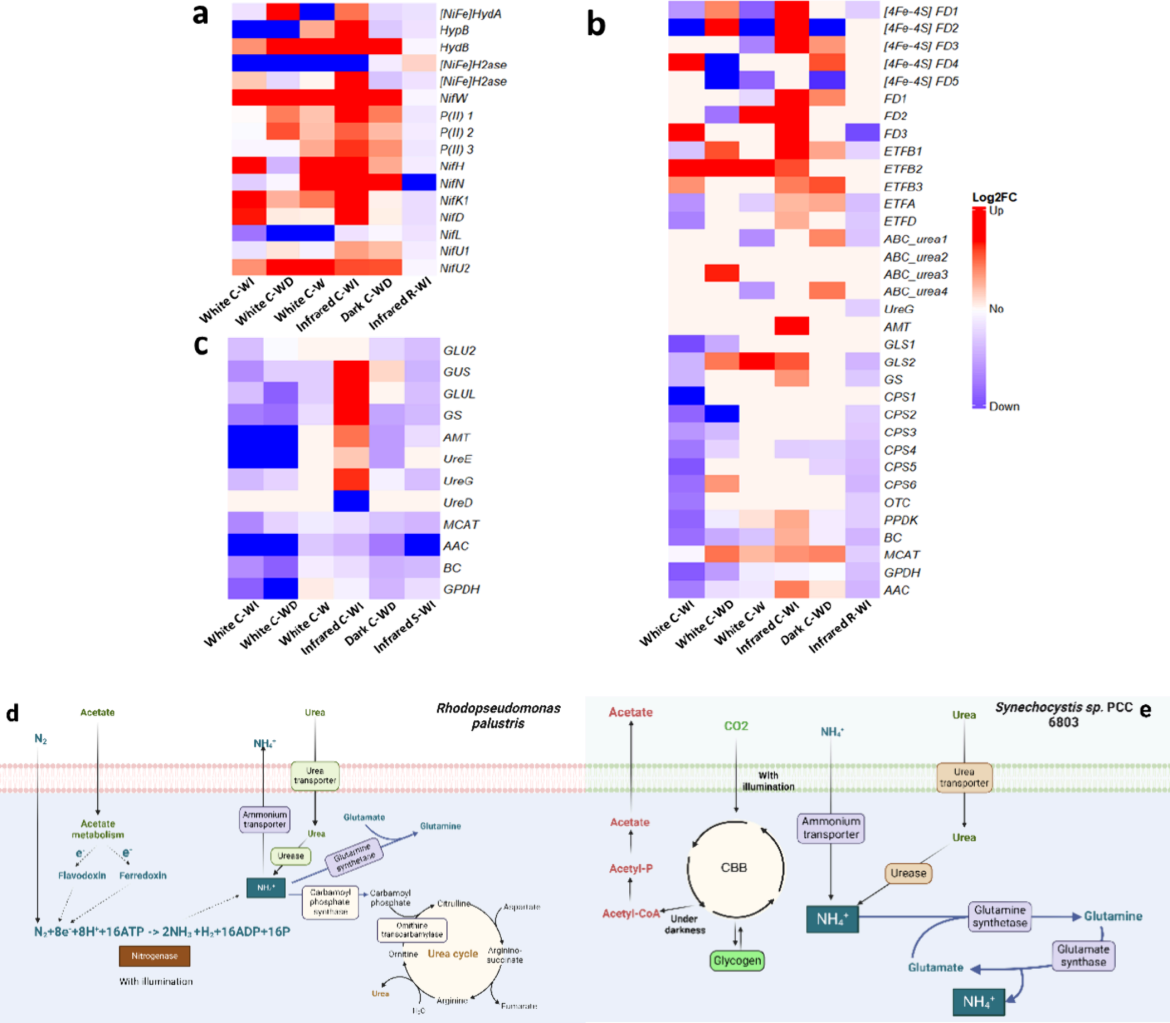
Heatmaps of relative abundance changes of (a) nitrogenase- and hydrogenase-related protein levels in *R. palustris*, (b) electron transport-, urea metabolism-, and ammonium metabolism-related protein levels in *R. palustris*, (c) urea and ammonium metabolism-related protein levels in *Synechocystis_acs*, and metabolic pathways of (d) nitrogen fixation, urea, ammonium, and fatty acid metabolism in *R. palustris*, and (e) urea, ammonium, and fatty acid metabolism in *Synechocystis_acs*. Up- and downregulated proteins (calculated by log 2 fold change compared to the control group. All results of *R. palustris* were compared to the white light period of a monoculture of *R. palustris* under light-infrared circadian illumination conditions. All results of *Synechocystis_acs* were compared to the white light period of monoculture of *Synechocystis_acs* under light-infrared circadian illumination conditions, indicated in red and blue, respectively. White: white light period; dark: dark period; infrared: infrared period; R: *R. palustris*; S: *Synechocystis_acs*; -W: constant white light illumination; -WD: circadian illumination of light and dark; -WI: circadian illumination of light and infrared.

In total, 11 nitrogenase- and nitrogen-fixation-related proteins, as well as 5 hydrogenase-related proteins, were detected (Figure 6). Proteomics demonstrated that light conditions significantly regulated the protein expression related to nitrogen fixation and hydrogen production. *R. palustris* possesses three different types of nitrogenases responsible for nitrogen fixation.^25^ In the group of C-WI, infrared illumination significantly stimulated the expression of molybdenum nitrogenases encoded by the *nif* genes and the nitrogen regulation proteins, leading to an obligatory production of hydrogen gas.^35^ Among the three classes of nitrogenases, the molybdenum counterpart functions most efficiently.^36^ Notably, during the white light illumination, the expressions of all proteins encoding these functions were downregulated or exhibited no significant difference compared to those in the control (R-WI), resulting in only a significant H_2_ production in C-WI during infrared phases. Nevertheless, with the upregulation of nitrogenase, a significant upregulation of the nickel-dependent hydrogenase ([NiFe] HydA) was also observed. As an enzyme that catalyzes the reversible oxidation of molecular H_2_, the produced H_2_ could be uptaken and consumed by the hydrogenase to supply reductants for nitrogen fixation or donate electrons for phototrophic growth (with sodium bicarbonate) when lacking favorable organic carbon sources like acetate,^35^ leading to a decrease of accumulated H_2_ gas (Figure 2a). On day 4, depleting levels of acetate were observed in C-WI treatment (Figure 1c). The [NiFe] HydA showed significant upregulation during infrared illumination and downregulation during white illumination in C-WI, suggesting that hydrogen uptake primarily occurred during infrared illumination. Given the extensive upregulation of hydrogenases, including HydA, HypB, and HydB (up to over 50,000-fold increase), the balance between hydrogen production and consumption appears to be heavily shifted toward consumption, thereby reducing net hydrogen accumulation (Figure 2a).

In contrast, in the group of C-WD, the nitrogenase molybdenum–iron protein (NifD), nitrogen-fixing protein (NifU), nitrogen regulatory protein P(II), and hydrogenase (NiFe) exhibited significant upregulation during white illumination, indicating that H_2_ production and uptake predominantly occurred during white illumination. This result highlights the superior light-harvesting efficiency of *R. palustris* under C-WD compared to C-WI. The dark phase appears to facilitate the synthesis of nitrogenase-related proteins, as evidenced by the significant upregulation of nitrogenase biosynthesis protein NifN and NifW.^37,38^ Compared to C-WI, the overall weaker induction of nitrogen-fixation proteins in C-WD may have resulted in a lower production of H_2_ than in the C-WI group (Figure 2a). Nevertheless, with constant illumination (C-W), most nitrogenase- and hydrogenase-related proteins were downregulated (*p* < 0.05, Figure 6), likely due to high oxygen levels in the system (Figure 2b). Consequently, no H_2_ production was detected.

In *R. palustris*, the electrons obtained from organic acids are first transported to ferredoxin or flavodoxin (Figure 6d),^14^ and then, the low-potential electrons from ferredoxin or flavodoxin will be further supplied to the nitrogenase for nitrogen fixation and hydrogen production.^39^ Ferrodoxin serves as a critical redox flux regulator among major metabolic pathways.^40^ In the treatments of C-WI and C-WD, the protein levels of both ferredoxin and flavodoxin were observed to be significantly upregulated (Figure 6b). As expected, the conditions of infrared and white-light illumination resulted in the highest response of electron transport proteins in the corresponding groups of C-WI and C-WD, respectively. This supported a highly promoted electron transfer toward the nitrogen fixation process during illumination, especially under the infrared condition (Figure 6b).

Urea and nitrogen gas served as major nitrogen sources for *R. palustris* in this study. On day 4, urea was detected in the supernatant of both monocultures (R-WI and Sacs-WI). However, it was depleted in all three cocultures (Figure 4), leading to an overall lower protein expression of urea transport in both strains (*R. palustris* and *Synechocystis_acs,*Figure 6b,c) and a reduced urea metabolic cycle in *R. palustris* than the control group (Figure 6b,d). Interestingly, while urea was depleted, proteins related to glutamate and glutamine synthase were significantly downregulated in the C-WI group during the light period. In contrast, infrared illumination significantly upregulated these proteins. This implies that *R. palustris* may exhibit either lower competitiveness for light harvesting under the visible light spectrum compared to *Synechocystis_acs*, or that nitrogenase activity was inhibited by the elevated oxygen levels during white light illumination, potentially allowing for an enhanced nitrogen fixation during the infrared period.

For *Synechocystis_acs*, urea was the only abiotic nitrogen source. Facing urea depletion in the medium, all ureases, glutamine synthetase, and glutamate synthase were expected to be downregulated (Figure 6e). Nevertheless, in response to infrared illumination (dark C-WI, Figure 6c), a dramatically upregulated ammonium transport protein (AMT), glutamine synthetase, and glutamate synthase were observed, indicating a potential ammonium supplier in the coculture. Taking *R. palustris* into consideration, proteomics results confirmed activated nitrogen fixation under an infrared illumination. This led to obligatory ammonium production in the cells of *R. palustris*. Additionally, an upregulation of the ammonium transporter (AMT) was detected in *R. palustris* during the same period (Figure 6b), implying a potential exchange of ammonium from *R. palustris* to *Synechocystis_acs* in the coculture. Previous studies revealed that the activity of nitrogenase could be inhibited by ammonium accumulation.^10^ Thus, the coculture established a symbiotic relationship in which *Synechocystis_acs* fixes carbon dioxide and supplies acetate to *R. palustris*, while *R. palustris* fixes nitrogen gas and provides ammonium in return. Through timely consumption of ammonium by *Synechocystis_acs*, the nitrogenase activity of *R. palustris* can be further promoted.

The enzyme acetyl-CoA carboxylase (ACC) is the first step in de novo fatty acid synthesis to catalyze carboxylation of acetyl-CoA to produce malonyl-CoA.^41^ In the coculture under circadian white and infrared illumination (C-WI), there was substantial variation of ACC expression, with a significant downregulation observed during the white light phase (White C-WI) and a significant upregulation during the infrared phase (infrared C-WI) in *R. palustris*. Meanwhile, in *Synechocystis_acs*, coculture with constant white illumination showed the least downregulation compared to the control among all cocultures. In contrast, cocultures under other illumination conditions (C–W and C-WD) demonstrated no significant difference in *R. palustris* compared with the control group. As a subunit of ACC, biotin carboxylase (BC) catalyzes the ATP-dependent carboxylation of biotin during fatty acid synthesis.^41^ The only upregulation of BC was observed in C-WI during infrared illumination from *R. palustris*. In the subsequent step, the produced malonyl-CoA was utilized by the enzyme malonyl-CoA-acyl carrier protein transacylase (MCAT) for fatty acid biosynthesis.^41^ In *Synechocystis_acs*, only C-W demonstrated no significant difference compared with the control, while all other treatments exhibited significant downregulation. In *R. palustris*, both C-W and infrared C-WI demonstrated significant upregulation. Additionally, the enzyme pyruvate phosphate dikinase (PPDK)^42^ and glycerol-3 phosphate dehydrogenase,^43^ which are positively correlated with fatty acid synthesis, also showed upregulation or minimal downregulation compared to the control group in C-W and infrared C-WI treatments. These results indicate that *R. palustris* contributed the most to fatty acid accumulation during infrared illumination in the C-WI group, while both *Synechocystis_acs* and *R. palustris* contributed to the increased fatty acid content in the C-W group. The significant difference in protein expression related to fatty acid biosynthesis during the illumination switch may help in maintaining the redox balance during photoheterotrophic growth.^43^

### 3.5 Environmental Implications

Phototrophic microorganisms hold great promise for capturing solar energy and converting greenhouse gases into sustainable energy carriers, providing an alternative solution to the increasingly fierce energy challenge. Communities of such phototrophs could even enable more diverse metabolic activities than single cultures; however, successful construction, control, and in-depth characterizations have rarely been reported to date. In this study, we combined strain engineering and light quality regulation to enable the coculture of cyanobacteria with purple nonsulfur bacteria toward clean production of energy carriers, e.g., H_2_ and fatty acids. The system was successfully constructed with both CO_2_ and N_2_ fixation. The industrial Haber-Bosch process for nitrogen fixation is one of the most energy-consuming and CO_2_-emitting processes of mankind.^44^ Therefore, the biotechnology proposed in this study that relies on atmospheric N_2_ and CO_2_ gas fixation will enable a more sustainable process and may be an important stepping stone toward a net-zero emissions economy.

Nevertheless, obstacles remain to be overcome before real applications can be developed. Inactivating the uptake hydrogenases in both strains could help prevent the consumption of produced H_2_,^45^ as observed in this study. Additionally, continuous reactors allowing for a constant removal of H_2_ and O_2_ using for instance selective membranes might be a technical solution to further improve the performance and finally long-term stability of N_2_ and CO_2_ fixation remains to be evaluated.

## Supporting Information Available

Strain engineering process; fatty acid calculation; gene ontology (GO) analysis; culture media compositions; eluent and temperature gradient for IC, HPLC, GCMS, and nano-LC; sulfur balance; and ammonium accumulation (PDF)
