# Supplementary material for: Engineering a Photoautotrophic Microbial Coculture toward Enhanced Biohydrogen Production
Source: Environ Sci Technol. 2024 Dec 12;59(1):337–48. doi: 10.1021/acs.est.4c08629 (PMC11741097; doi:10.1021/acs.est.4c08629)
Supplement: Supplementary file 1 — es4c08629_si_001.pdf [file es4c08629_si_001.pdf]

Supplementary information

Engineering a photoautotrophic microbial coculture toward enhanced biohydrogen production

Minmin Pan[a], Rodrigo Amarante Colpo[a], Stamatina Roussou[b], Chang Ding[c], Peter Lindblad[b], Jens O. Krömer[a,*]

[a]Department of Microbial Biotechnology, Helmholtz Centre for Environmental Research - UFZ, Leipzig, 04318, Germany

[b]Microbial Chemistry, Department of Chemistry-Ångström, Uppsala University, Box 523, 751 20 Uppsala, Sweden

[c]Department of Molecular Environmental Biotechnology, Helmholtz Centre for Environmental Research - UFZ, Leipzig, 04318, Germany

**Corresponding author*: jens.kroemer@ufz.de (J. Krömer)

Totally 11 pages including 2 figures, and 10 tables

Supplementary text 1. Engineered strain *Synechocystis_acs*

1.1 Cultivation conditions of *Escherichia coli* and *Synechocystis* PCC 6803

The *Escherichia coli* (*E. coli*) strains were cultivated at 37 °C using LB medium either in liquid or on plates containing 1.5 % agar (w/v). *Synechocystis* PCC 6803 (*Synechocystis*) strains were grown at 30 °C under the illumination of 65 μmol photons $m^{-2}s^{-1}$ in BG11 medium, both in liquid cultures and 1.5 % agar (w/v) plates. Kanamycin (Sigma-Aldrich) was added to the media for both organisms at a final concentration of 50 μg/ml when necessary.

1.2 Plasmid construction and transformation to *Synechocystis*

The plasmid for the production of WT_PKPa_Δacs, was based on a pEERM vector (Englund *et al.,* 2015). The method relies on homologous recombination using 1000bp homologous arms upstream and downstream of the acs locus. The sequences to be used were amplified from the *Synechocystis* genome and then joined with the phosphoketolase gene (PKPa) under the control of the PtrcRiboJ promoter (Liu *et al.,* 2019) and the kanamycin cassette through overlap extension PCR. The final construct was then ligated with the backbone vector through linear ligation. The backbone vector and the final product were phosphorylated in either the 5' or the 3' DNA end and ligated together. The ligation product was transformed to competent *E. coli* T7 cells (NEB) and successful clones were identified via colony PCR and confirmed by sequencing.

Natural transformation was used to engineer the cyanobacterium genome with the correct plasmid. *Synechocystis* wild-type strain was cultivated to OD_{730} = 0.5–1.2 in liquid BG11. The cells were harvested by centrifugation at 5000 g for 10 minutes, washed twice with fresh BG11, and resuspended to an OD730 of 2.5 in 400 μl of BG11. This suspension was mixed with the plasmid to a final concentration of 10 μg μl^{-1} and incubated under low light at 30 °C for 4-5 hours. Following incubation, cells were spread

on membrane filters placed on BG11 plates without antibiotics. The next day, the filters were transferred to BG11 plates containing the selection antibiotic(s). Isolated colonies appeared after 7-10 days, which were then regrown on fresh plates and screened by colony PCR. Verified positive clones were further cultivated in 6-well tissue culture plates (Sarstedt) with kanamycin to ensure complete integration of the desired genetic construct into the genome. Successful integration was confirmed through PCR using specific primers.

Table S1.1: Engineered strain of *Synechocystis* and integration vector

**PKPa* originated from *Pseudomonas aeruginosa*

Amplification of homologous recombination regions	
US_acs_F	/5Pho/TTTCCACTTCACTTGGTT
US_acs_Ptrc_R	GAGCGCTCACAATTGTCAACAGCTCGGTTCTCCGTCAAAGTCT
DS_acs_KmR_F	ACACTGGCAGAGCATTACGCTGACTTAGCGTGTTGGACAAATT
DS_acs_R	CAAATTAGCCAAACCCAC

Table S1.2: Primers used in this study

Synechocystis strain/plasmid	Relevant Genotype	References
WT_PKPa_Δacs	Δacs:: (P*trc*RiboJ-*PKPa**-T)Km^R	This study
IVE Δacs-PKPa	Km/ PtrcRiboJ-PKPa-T targeting the *acs* (*sll0542*)	This study

Amplification of promoters, terminators, replication origins and antibiotic cassettes	
Ptrc_F	/5Phos/GAGCTGTTGACAATTGTGAGCG
Ptrc_acs_F	CCATTAAAGACTTTGACGGAGAACCGAGCTGTTGACAATTGTG
Ptrc_RiboJ_R	CATTTTTTTCCTCCTTCTAGT
PtrcRiboJ_pkpa_R	CTGAGGATGACTCCACATCATTTTTTTCCTCCTTCT
TB15_F	GCAGCCAGGCATCAAATAA

TB15_PKPa_R	CCCGCCTTATTGCGTTAATGCAGCCAGGCATCAAAT
IVE_F	CTCATGACCAAAATCCCT
IVE_R	/5Phos/CTGGCAGTTCCCTACTCT
KmR_TB15_F	GGCCTTTCTGCGTATACTAGTTATATTTATACTCGAGCTGATCC
KmR_overlap_F	GGCCTTTCTGCGTATACT
KmR_acs_R	CTCCCGTAATTTGTCCAACACGCTAAGTCAGCGTAATGCTCTG
KmR_F	GGTTGCATTCGATTCCTGTT
KmR_DS_acs_R	CTCCCGTAATTTGTCCAACACGCTAAGTCAGCGTAATGCTCTG

Amplification of endogenous/codon optimized genes	
PKPa_PtrcRiboJ_F	TAGAAGGAGGAAAAAAATGATGTGGAGTCATCCTCAG
PKPa _TB15_R	ATTTGATGCCTGGCTGCATTAACGCAATAAGGCGGG

E. coli colony PCR and sequencing primers	
pkpa_mid_R	CGGTATGGGGATTCACATG
pkpa_seq_R	CAGGCGGACACCACGGCTTCT
IVE_colony_F	GGCCTTTTGCTCACATGTTC
IVE_colony_R	CTTTTCTACGGGGTCTGACG
US_acs_colony_R	GGACAGTGGGGAAAAGATCA
DS_acs_colony_R	TTTCTTCATCCGTGGGACTC

Synechocystis colony PCR to investigation of genomic integration and fully segregation	
UUS_acs_F	ACATCGGCTGGATGATGTTT
DDS_ acs_F	GGGCTTTGTTATTGGCTGAG

Primers to investigate fully segregation on *Synechocystis* genome	
acs_integr_F	AGCAATCCGGAGAAGTTT
acs_integr_R	AATAAAGGCTCGGATGGC

Supplymentary text 2. Calculation of fatty acid titer and content

The fatty acid data from GC-MS measurements were normalized to the internal standard nonadecanoic acid. The normalized data were then calculated using the fatty acid titer ($mg_{fatty\ acid}$ L^{-1}) according to the equations listed below.

$$\mathrm{W\_C8{:}0} = \frac{S + 3 \times 10^6}{2 \times 10^8} \tag{S1}$$

$$\mathrm{W\_C12{:}0} = \frac{S - 998425}{1 \times 10^8} \tag{S2}$$

$$\mathrm{W\_C14{:}0} = \frac{S - 756977}{1 \times 10^8} \tag{S3}$$

$$\mathrm{W\_C16{:}0} = \frac{S - 3 \times 10^6}{2 \times 10^8} \tag{S4}$$

$$\mathrm{W\_C16{:}1} = \frac{S + 3 \times 10^6}{1 \times 10^8} \tag{S5}$$

$$\mathrm{W\_C18{:}0} = \frac{S + 1 \times 10^6}{2 \times 10^8} \tag{S6}$$

$$\mathrm{W\_C18{:}1} = \frac{S + 891818}{2 \times 10^8} \tag{S7}$$

$$\mathrm{W\_C18{:}2} = \frac{S + 3 \times 10^6}{1 \times 10^8} \tag{S8}$$

$$\mathrm{W\_C20{:}0} = \frac{S + 2 \times 10^6}{2 \times 10^8} \tag{S9}$$

$$\mathrm{W\_C22{:}1} = \frac{S + 2 \times 10^6}{1 \times 10^8} \tag{S10}$$

Where, W_ is the fatty acid titer (mg L^{-1}), C represents specific fatty acid, S is the measuring intensity by GC-MS. Afterwards, the fatty acid contents were calculated by dividing the corresponding total biomass titer (listed in table below) at day 4.

	Synechocystis (mg L^{-1})	*R. palustris* (mg L^{-1})	Sum (mg L^{-1})

R+Sacs-LR	118.78	154.18	272.97
R+Sacs-LD	163.80	86.94	250.74
R+Sacs-L	289.51	60.88	350.39
R-LR	0	41.23	41.23
Sacs-LR	319.59	0	319.59

Supplementary text 3. Analysis of Gene Ontology (GO) and differentially expressed proteins

The method adopted in the present study is a novel approach to integrate the Gene Ontology term enrichment analysis with differentially expressed proteins and enrichment metrics. Specifically, the proteomics data was classified in its comprehensive functional groups by the databases UniProt and Gene Ontology. Firstly, extract the protein IDs from the annotated genomes of the two strains and map them to UniProt IDs. Then, utilized Fisher´s method for combining p-values from independent measurements of protein abundance associated with the same GO annotation, identifying significant average differential expressions. The Benjamini-Hochberg procedure was further applied to adjust p-values, effectively controlling the false discovery rate (FDR). Followed by the hypergeometric test, the overrepresentation of GO terms among the set of differentially expressed genes (proteins) was filtered and shown as enrichment detection.

Table. S2 Composition of the modified M27 growth/preculture medium

Components	Concentration (mg L^{-1})
KH_2PO_4	500
$MgSO_4$ x $7H_2O$	400
NaCl	400

CaCl x $2H_2O$	50
$C_3H_{10}ClNO_3S$	120
$C_6H_5FeO_7$	5
Viatmin B12	0.04
$C_2H_3NaO_2$ (only for preculture)	500
CH_4N_2O (only for growth medium)	60
$NaHCO_3$ (only for growth medium)	820
ZnSO4 x 7H2O	0.1
MnCl2 x 4H2O	0.03
H3BO3	0.3
CoCl2 x 6H2O	0.2
CuCl2 x 2H2O	0.01
NiCl2 x 6H2O	0.02
Na2MoO4 x 2H2O	0.03

Table. S3 Composition of the BG11 growth medium

Components	Concentration (mg L^{-1})
$NaNO_3$	1500
K_2HPO_4	40
$MgSO_4{\cdot}7H_2O$	75
$CaCl_2{\cdot}2H_2O$	36
Citric acid	6
Ferric ammonium citrate	6
Na_2EDTA	1
Na_2CO_3	20
H_3BO_3	2.86
$MnCl_2{\cdot}4H_2O$	1.81

$ZnSO_4 \cdot 7H_2O$	0.22
$Na_2MoO_4 \cdot 2H_2O$	0.39
$CuSO_4 \cdot 5H_2O$	0.08
$Co(NO_3)_2 \cdot 6H_2O$	0.05

Table. S4 Eluent gradient of cation chromatograph (Integrion)

Time (min)	Concentration of Methanesulfonic Acid (EGC500, cation, mM)
0	1.5
15	1.5
20	15
22	15
23	3
30	1

Table. S5 Eluent gradient of ion chromatograph (ICS-6000)

Time (min)	Concentration of KOH (mM)
0	1
5	1
14	10
23	20
38	60
48	80
55	80
55	1
60	1

Table. S6 Eluent gradient of HPLC

Time (min)	B (%)	Flow (ml/min)
0	100	0.4
20.5	45	0.4
22	45	0.4
23	100	0.4
31	Stop run	

Table. S7 Temperature gradient of GCMS

Time (min)	Rate (°C /min)	Target value (°C)	Hold time (min)
0			
1	0	60	1
37.5	10	325	10
41.15	100	60	1
42		Stop run	

Table. S8 nano-LC eluent gradient for proteomics

Time (min)	Flow (μL/min)	B (%)
0	0.3	4
1	0.3	4
5	0.3	10
100	0.3	35
120	0.3	55
130	0.3	90
135	0.3	90
137	0.3	4

145	0.3	4

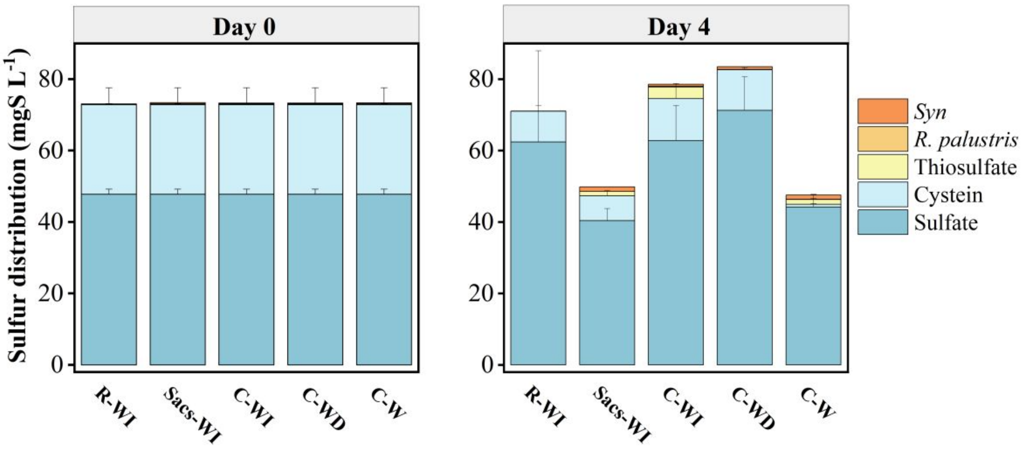

Fig. S1 Sulfur balance calculation in treatments at day 0 and day 4

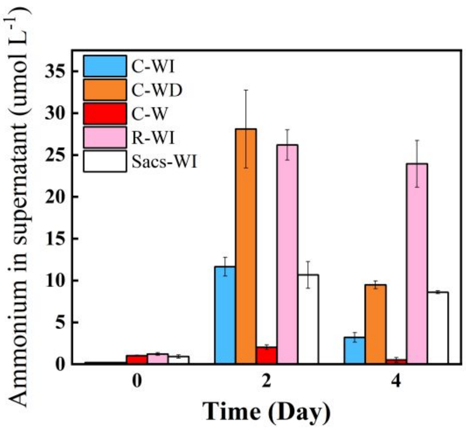

Fig. S2 Ammonium accumulation in supernatant
